# Insulin resistance, leptin and adiponectin in lean and hypothyroid children and adolescents with obesity

**DOI:** 10.1186/s12887-022-03318-x

**Published:** 2022-05-02

**Authors:** Doaa El Amrousy, Dalia El-Afify, Shaimaa Salah

**Affiliations:** 1 Pediatric Department, Faculty of Medicine, Tanta University, Tanta, Egypt; 2 Department of Clinical Pharmacy, Faculty of Pharmacy, Tanta University, Tanta, Egypt; 3 Pediatric Department, Faculty of Medicine, Kafrelsheikh University, Kafrelsheikh, Egypt

**Keywords:** Hypothyroidism, Insulin resistance, Leptin, Adiponectin, Obesity

## Abstract

**Background:**

Obesity usually complicates hypothyroidism. Adipokines like leptin and adiponectin secreted by adipose tissue modulate insulin resistance (IR), appetite, and obesity. The association between adipokines, IR, and thyroid hormone has not been sufficiently studied in children. We investigated leptin and adiponectin as well as IR and their association with thyroid hormone in both lean and hypothyroid children and adolescents with obesity.

**Methods:**

The study included 30 lean hypothyroid, 30 hypothyroid children and adolescents with obesity, and 30 healthy lean children as the control group. Serum thyroid-stimulating hormone (TSH), free triiodothyronine (fT3), free thyroxine (fT4), fasting blood glucose, fasting insulin, homeostatic model assessment method of insulin resistance (HOMA-IR), leptin, and adiponectin levels were estimated in all participants.

**Results:**

Fasting insulin, HOMA-IR, and leptin levels were significantly elevated in hypothyroid children compared to the control group; more in hypothyroid children with obesity. In contrast, adiponectin levels were significantly lower in the hypothyroid children with obesity compared to the lean hypothyroid children and controls. HOMA-IR was positively correlated to TSH and BMI but inversely correlated with fT3 and fT4 in hypothyroid children. There was no correlation between IR and either leptin or adiponectin levels. Leptin and adiponectin levels correlated well with BMI in hypothyroid children and adolescents with obesity.

**Conclusion:**

Insulin resistance and leptin levels are increased in hypothyroid children and adolescents; more in those with obesity. IR is not related to leptin and adiponectin levels, however, leptin and adiponectin levels correlate well with BMI in hypothyroid children and adolescents with obesity.

**Impact:**

Insulin resistance (IR) and leptin levels increase in hypothyroid children and adolescent; more with obesity. IR is not related to leptin and adiponectin levels, however leptin and adiponectin levels correlated well with BMI in hypothyroid children and adolescents with obesity.

## Introduction

The thyroid hormone regulates the body’s basal metabolic rate and heat production. It also affects the metabolism of protein, carbohydrates, and lipids. Thyroid disorders affect the metabolic processes and cause changes in appetite, muscle mass, body weight, and adipose tissue. Thyroid diseases have also been linked to cardiovascular diseases, insulin resistance (IR), and type 2 diabetes mellitus [1, 2].

Hypothyroidism is characterized by a decrease in free triiodothyronine (fT3) and free thyroxine (fT4) levels and an increased level of thyroid-stimulating hormone (TSH) which is usually associated with obesity and IR [3]. Hypothyroidism decreases the basal metabolic rate, oxygen consumption, and lipolysis and it may affect the action of adipose tissue [4].

Adipose tissue produces several adipokines, including leptin and adiponectin that play a role in energy balance regulation and affect cellular metabolic functions. Leptin controls food intake and maintains energy balance. Its levels increase in obesity and is correlated with IR [5]. Adiponectin mediates anti-atherosclerotic, anti-inflammatory, and anti-hyperglycemic actions, and in contrast to leptin, its level decreases in cases of IR and obesity [6].

Insulin resistance has been observed in adult patients with hypothyroidism in previous studies [7–9]. However, the association between adipokines, IR, and thyroid hormones has not been well studied in children. In this study, we investigated leptin and adiponectin as well as IR (HOMA-IR) and their associations with thyroid hormone in both lean and hypothyroid children and adolescents with obesity.

## Materials and methods

This cross-sectional study was conducted on 30 lean hypothyroid and 30 hypothyroid children and adolescents with obesity attending the outpatient endocrinology clinic of the Pediatric Department of Tanta and Kafrelsheikh University Hospitals during the period from January 2018 to April 2021. Thirty healthy lean children and adolescents of matched age and sex served as the control group. The study was approved by the Faculty of Medicine, Tanta University ethical committee, and informed consent was obtained from the parents of all participants. The study was performed in accordance with the ethical standards of the institutional research committee and with the 1964 Helsinki declaration and its later amendments.

The inclusion criteria were: age less than 18 years and newly diagnosed overt hypothyroidism.

The exclusion criteria were: acute or chronic inflammatory diseases, liver, renal, autoimmune diseases, diabetes mellitus, or any other endocrine diseases.

Hypothyroidism was diagnosed in the presence of high TSH and low fT3 and fT4 levels. Children and adolescents with hypothyroidism were subdivided into two groups:

Group I: included 30 hypothyroid children and adolescents with normal body mass index (BMI) that ranged from the 5th to 85th percentile for age and sex.

Group II: included 30 hypothyroid children and adolescents with a BMI greater than the 95th percentile for their age and sex [10].

A complete physical examination and medical history were carried out for all participants. Anthropometric measurements in the form of weight, height, and BMI were performed for all included children. Tanner staging was used to assess and classify pubertal staging. Therefore, individuals were considered prepubertal when they were at stage 1 and pubertal when they were at stages 2, 3, 4, or 5.

Venous blood samples after at least 8 h overnight fasting were obtained from all participants to assess:

1-Thyroid function: Serum TSH, fT3, and fT4 concentrations were measured by immunoassay using commercial kits (Bioassay Technology Laboratory, Shanghai, China).

2- Fasting blood glucose (FBG) assay was carried out using the colorimetric glucose oxidase method by reagent kits (Spinreact, Santa Coloma, Spain).

3- Fasting insulin measurement was carried out using commercial enzyme-linked immunosorbent assay (ELISA) kits (DRG International Inc., USA).

The homeostatic model assessment method of insulin resistance (HOMA-IR) was calculated using the following formula: HOMA-IR = fasting serum glucose (mmol/L) × fasting serum insulin (μU/mL)/ 11.5.

4- Serum leptin and adiponectin assays were carried out using commercial ELISA assay kits (Ray Biotech Inc., Norcross, USA).

### Statistical analysis

Data were analyzed using SPSS software version 11. Quantitative data were presented as mean ± standard deviation (SD), while qualitative data were presented in the form of numbers and percentages. Comparison of quantitative data between the three groups were carried out using one way analysis of variance (ANOVA) test followed by Tukey-Kramer test. Chi square test was used to assess any significant difference in qualitative data between the three studied groups. Correlation between HOMA-IR, leptin, adiponectin, BMI, and thyroid hormone was evaluated using Persons correlation coefficient. A *p-*value < 0.05 was considered statistically significant.

## Results

The study included 30 lean hypothyroid children and adolescents with a mean age of 11 years, 30 hypothyroid children and adolescents with obesity with a mean age of 10.6 years, and 30 healthy lean children and adolescents with a mean age of 10. 8 years. Table 1 shows the demographic and clinical data of the three studied groups. There were no significant differences between the three groups in regards to the age, sex, height, pubertal development, or fasting blood glucose. Weight and BMI were significantly higher in hypothyroid children with obesity compared to lean hypothyroid children and controls (*P* < 0.001). Concerning thyroid hormone, TSH was significantly higher in both lean and hypothyroid children with obesity compared to the controls, while fT3 and fT4 were significantly lower in lean and hypothyroid children with obesity compared to the control group.

**Table 1:** Clinical and demographic data of the participants

Parameters	Lean hypothyroid group (*n* = 30)	hypothyroid with obesity group (*n =* 30)	Control group (*n =* 30)	*p*-value
Age (years)	11 ± 2.17	10.6 ± 2.1	10. 8±2. 13	0. 8
Gender (M/F)	11:19	10:20	12:18	0.87
Weight (Kg)	40 ± 9	57± 12.5^a,b^	39. 8± 7. 8	< 0.001
Height (cm)	140± 12.2	137 ± 10.5	141± 12. 8	0.78
BMI (Kg\m^2^)	20.2 ± 4.1	30 ± 4.3 ^a,b^	19.9 ± 2.2	< 0.001
Pubertal development				
-Prepubertal	18 (60%)	16 (53.3%)	15 (50%)	0.651
Pubertal	12 (40%)	14 (46. 7%)	15 (15%)	
TSH (mU\L)	22.4± 7.1^a^	26.2 ± 9.6^a^	2. 8±0.67	< 0.001
fT3 (ng\dl)	2. 7±0.5^a^	2.5 ± 0.68^a^	3. 8±0.83	< 0.001
fT4 (μgl\dl)	8. 7±1.1^a^	8.1 ± 1.7^a^	18 ± 3.5	< 0.001
FBG (mg\dl)	83 ± 5.3	84 ± 5.4	82 ± 5.4	0.34
FBI (μIU\ml)	9.3 ± 2.3^a^	11.4 ± 2.4^a,b^	4 ± 0. 8	< 0.001
HOMA-IR	1.9 ± 0.4^a^	2.3 ± 0.44^a,b^	0. 8±0.15	< 0.001
Leptin (ng\ml)	15.3 ± 2.1^a^	17.4 ± 2^a,b^	7.2 ± 1.2	< 0.001
Adiponectin (ng\ml)	12.1 ± 2.3	9. 8±2^a^	12±2	0.034

*M/F* Male to female, *BMI* Body mass index, *TSH* Thyroid stimulating hormone, *fT3* free triiodothyronine, *fT4* Free thyroxine, *FBG* Fasting blood glucose, *FBI* Fasting blood insulin, *HOMA-IR* Homeostasis model assessment method of insulin resistance

^a^: significant difference compared to the control group, b: significant difference compared to the lean hypothyroid group

Both lean and hypothyroid children with obesity had significantly higher levels of fasting insulin and HOMA-IR compared to the control group. In addition, fasting insulin and HOMA-IR were significantly higher in hypothyroid children with obesity compared to lean hypothyroid children (*P* < 0.001).

Regarding adipokines, serum leptin was significantly elevated in lean and hypothyroid children with obesity groups compared to the control group, more in hypothyroid children with obesity (*P* = 0.034).

In contrast, adiponectin levels were significantly lower in hypothyroid children with obesity compared to lean hypothyroid children and controls. Adiponectin levels were comparable in both lean hypothyroid children and the control group.

In hypothyroid children, there was a significant positive correlation between HOMA-IR and both TSH and BMI and a significant negative correlation with fT3 (r = − 0.74, *P* = 0.002) and fT4 (r = − 0.62, *P* = 0.003). However, there was a non-significant correlation between HOMA-IR and both leptin and adiponectin (Table 2).

**Table 2:** Correlation between HOMA-IR with clinical and laboratory data in hypothyroid children

Parameters	HOMA-IR	
	r	*p-*value
Age	0.02	0.92
Weight	0.1	0.6
BMI	0.73	0.003^a^
TSH	0.63	0.017^a^
fT3	− 0.74	0.002^a^
fT4	− 0.62	0.003^a^
Leptin	0.29	0. 12
Adiponectin	− 0.2	0.29

*BMI* Body mass index, *TSH* Thyroid stimulating hormone, *fT3* Free triiodothyronine, *fT4* Free thyroxine, *FBG* Fasting blood glucose, *HOMA IR* Homeostasis model assessment method of insulin resistance

^a^ means significant *p*-value< 0.05

Leptin showed a significant positive correlation with TSH (r = 0.51, *P* = 0.004) and a significant negative correlation with fT4 (r = − 0.42, *P* = 0.02) in lean hypothyroid children. While in hypothyroid children with obesity, leptin showed a significant positive correlation with BMI (r = 0.51, P = 0.004) and a significant negative correlation with adiponectin (r = − 0.44, *P* = 0.015). There was no significant correlation between leptin and adiponectin with TSH, fT3, and fT4 in hypothyroid children with obesity (Table 3).

**Table 3:** Correlation of leptin and adiponectin with clinical and laboratory data of lean and hypothyroid children with obesity

Parameters	Lean hypothyroid group				hypothyroid children with obesity group			
	Leptin		Adiponectin		Leptin		Adiponectin	
	r	p	r	p	r	p	r	p
Age	0.18	0.37	− 0.3	0.1	0.02	0.92	0. 13	0.42
Weight	0. 13	0.49	− 0.19	0.31	0.25	0. 16	0.27	0.15
BMI	0.11	0.56	− 0.2	0.29	0.51	0.004^a^	− 0.44	0.015^a^
TSH	0.51	0.004^a^	0.02	0.92	0.17	0.34	−0.347	0.06
fT3	-0. 22	0.24	−0.06	0.75	−0.32	0.08	0.29	0. 12
fT4	− 0.42	0.02^a^	-0. 13	0.48	−0.17	0.37	0.23	0. 22

BMI: body mass index, TSH: Thyroid stimulating hormone fT3: free triiodothyronine, fT4: free thyroxine, FBG: fasting blood glucose

^a^ means significant *p-*value< 0.05

## Discussion

In the present study, we assessed HOMA-IR, leptin, and adiponectin in lean and hypothyroid children and adolescents with obesity, and we also studied their association with thyroid hormone in such children.

In the present study, HOMA-IR and fasting blood insulin levels were significantly higher in hypothyroid children and adolescents with or without obesity compared to the control group, which is consistent with the results of previous studies indicating that hypothyroidism can induce IR and this IR is related to hypothyroidism more than BMI [7–9, 11–14].

Insulin resistance occurs when insulin’s biological effect on the skeletal muscle, liver, adipose tissue, and other peripheral tissues is less than its expected physiological effect. IR in hypothyroidism is suggested to be due to peripheral IR that occurs in skeletal muscle and adipose tissue due to the altered expression of glucose transporters [15]. Thyroid hormone deficiency is also associated with alteration in the peripheral blood flow and may cause changes in glucose transporters in the cellular plasma membrane which plays a role in the development of IR [9, 16, 17].

HOMA-IR was significantly positively correlated with TSH but negatively correlated with fT3 and fT4 which was reported in other studies [18–23]. These results may emphasize the fact that changes in TSH, fT3, and fTT4 can develop IR in hypothyroid children.

Moreover, fasting insulin level and HOMA-IR were significantly elevated in hypothyroid children and adolescents with obesity compared to lean hypothyroid children and adolescents which reflects the additive role of obesity in the development of IR in hypothyroid children with obesity. Obesity can cause IR through several mechanisms, such as inhibition of carbohydrate metabolism through intracellular inhibition of insulin signaling, increased cytokines, and hormonal production by adipose tissue [24, 25].

Leptin levels were significantly higher in hypothyroid children and adolescents compared to the control group, which was in agreement with the results of previous studies [14, 16, 26, 27]. This was contradictory to the results obtained by Iglesias et al. [28] who found that leptin was lower in their hypothyroid patients and Corbetta et al. [29] who found normal leptin levels in hypothyroid patients.

Leptin and thyroid hormone affect each other as TSH stimulates TSH-receptors on the surface of adipocytes and can directly increase the production of leptin in adipose tissue [30–33]. On the other hand, leptin regulates the production of thyrotropin-releasing hormone (TRH) in the hypothalamus and increases the release of TSH from the anterior pituitary [32, 33]. This may explain the significant positive correlation between leptin and TSH in lean hypothyroid children and adolescents, which has also been reported by others [14, 30]. We also observed a significant negative correlation between leptin levels and fT4 in lean hypothyroid children and adolescents, which may be due to the ability of leptin to stimulate the conversion of T4 to T3 by regulating the activity of iodothyronine deiodinase [34]. The significant correlations between leptin with TSH and fT4 suggest that the changes in thyroid hormone are directly related to leptin independent of obesity.

The higher levels of leptin in our hypothyroid children and adolescents with obesity compared to lean hypothyroid patients and the presence of a significant positive correlation between leptin and BMI and the lack of a significant correlation between leptin and thyroid hormones in hypothyroid children and adolescents with obesity suggest that the relationship between leptin and thyroid hormone in hypothyroid children with obesity may be different and affected by additional factors related to obesity.

Interestingly, our study showed that adiponectin levels were comparable in both lean hypothyroid children and adolescents and healthy controls, which was in agreement with the results of other investigators [16, 28, 35]. However, adiponectin levels were significantly lower in hypothyroid children and adolescents with obesity compared to lean hypothyroid children and healthy controls, indicating that adiponectin levels are related to obesity more than hypothyroidism. Moreover, adiponectin levels in hypothyroid children and adolescents with obesity had a significant negative correlation with BMI, which may suggest that obesity can only affect adiponectin levels in such patients.

We also found a non-significant correlation between HOMA-IR and both leptin and adiponectin in hypothyroid children and adolescents, which was reported by others [14, 28, 35, 36]. This result suggests that the increase in IR in hypothyroid children may not be related to leptin or adiponectin.

The study has several limitations. First, there is a relatively small number of children included. Second, we didn’t include obese euthyroid children as another control group. Third, because of the cross-sectional study nature, the cause-effect relationship cannot be estimated. Further studies with a larger number of patients are required for investigation and clarification of the relationship between adipokines, IR, and thyroid hormones.

### Conclusion

IR and leptin are increased in hypothyroid children and adolescents; more in those with obesity. IR is not related to leptin and adiponectin levels, however, leptin and adiponectin levels correlate well with BMI in hypothyroid children and adolescents with obesity.

## Data Availability

The datasets generated and/or analyzed during the current study are not publicly available as we don’t have consent from our institutional to make patients’ data publicly avialable but the data are available from the corresponding author on reasonable request.
